# Interventions for Transition-Related Challenging Behavior in Individuals with Disabilities: A Targeted Research Synthesis and Meta-Analysis of Studies Published in Behavior Analytic Journals

**DOI:** 10.1177/01454455261421144

**Published:** 2026-03-04

**Authors:** Ji Young Kim, Vidhya Ravichandran, Mariola Moeyaert, Harley Ditzler, Maddux Ryan, Daniel M. Fienup

**Affiliations:** 1 Pennsylvania State University – Harrisburg, Middletown, PA, USA; 2 Widener University, Chester, PA, USA; 3 University at Albany, State University of New York, Albany, NY, USA; 4 Teachers College, Columbia University, New York, NY, USA

**Keywords:** intellectual and developmental disabilities, transitions, meta-analysis, intervention, challenging behavior

## Abstract

Transition-related challenging behavior is common among individuals with intellectual and developmental disabilities, particularly during changes between activities. Two hypothesized controlling variables—unpredictability and negative incentive shifts—may contribute to the occurrence of these behaviors. This targeted research synthesis and meta-analysis identified studies published in behavior-analytic journals examining interventions developed to decrease transition-related challenging behavior. Nineteen experiments across sixteen peer-reviewed articles were included in the targeted research synthesis, with interventions categorized as those incorporating a signaling stimulus (e.g., advance notice, visual schedules) or other procedures (e.g., differential reinforcement, extinction). A multilevel meta-analysis was conducted to estimate overall intervention effects and examine whether the intervention type moderated its effectiveness. Results indicated that, although overall effects were not statistically significant, consequence-based interventions without signaling stimuli were associated with greater reductions in challenging behavior. Implications for practice, including the integration of signaling stimulus and consequence-based procedures, are discussed, along with directions for future research on intervention efficiency, generalization, and long-term maintenance.

## Introduction

Individuals with intellectual and developmental disabilities (IDD) often experience behavioral, cognitive, and social challenges that increase the likelihood of engaging in challenging behaviors (National Institute of Health, 2024). Approximately 13% to 30% of young children engage in challenging behaviors that require some form of intervention, with those who have communication or social difficulties being particularly at higher risk (Horner et al., 2002; Strand et al., 2021). Common challenging behaviors in individuals with IDD such as verbal or physical aggression, self-injurious behaviors, tantrum behavior, and stereotypies, typically necessitate immediate intervention to address their impact (Matson et al., 2010; Tevis & Matson, 2022).

One common situation that can evoke challenging behavior is transitioning between activities. Transitions, defined as a period when one activity, event, or stimulus context ends and another begins (Luczynski & Rodriguez, 2015; Mitteer et al., 2023), are an important part of daily routines. For example, in a school, students must transition between multiple activities in a timely manner throughout the day to ensure effective learning (Ryan et al., 2019). Children with IDD often experience difficulty when transitioning between activities or environments, leading to challenging behaviors during the transition to resist change (Schreibman et al., 2000). Engaging in challenging behavior during transitions can be harmful to the individual displaying it and disruptive to others and may prevent the individual from experiencing similar educational and social opportunities compared to their peers. In a classroom setting, if a student engages in challenging behavior when transitioning between activities, it could interrupt their learning and distract their peers in the classroom. If the behavior occurs frequently, teachers may need to intervene, potentially separating the student from peers and further limiting their learning opportunities.

Two hypotheses have been suggested to explain why transitional activities may lead to challenging behavior. First, the transition occurs without prior notice and, therefore, includes an element of unpredictability (Brewer et al., 2014; Castillo et al., 2018). To mitigate the effects of unpredictability, interventions including a signaling stimulus such as a picture prompt or activity schedule have been used (e.g., McCord et al., 2001; Tustin, 1995). Interventions that include a signaling stimulus may involve providing the target individual advance notice, which is “any procedure used to signal when the current activity will end and what the next activity will be” (Brewer et al., 2014; p. 118), before a transition occurs to remove uncertainty. While the term signaling stimulus refers broadly to any antecedent stimulus used to signal the end of one activity and the onset of another (e.g., visual, verbal, or gestural cues), the term advance notice is one common type of signaling stimulus that provides information about the upcoming transition (e.g., a countdown, warning statement, or visual schedule). Conceptually, providing a stimulus that signals a change can be effective as it removes the motivation (i.e., establishing operation) for escape (Brewer et al., 2014).

Advance notice is typically regarded as an effective procedure; however, the existing body of literature shows mixed results when advance notice is used as an intervention for transition-related challenging behavior. Several studies demonstrated that advance notice was effective in decreasing challenging behaviors such as stereotypy (Tustin, 1995) and self-hitting (Flannery & Horner, 1994) during transitions. Reviews also showed that advanced notice procedures were promising at reducing transition-related challenging behavior for individuals with autism (Lequia et al., 2012; Sterling-Turner & Jordan, 2007). On the other hand, studies showed that advance notice did not reduce challenging behaviors such as tantrums (Wilder et al., 2006) and noncompliance in young children (Cote et al., 2005; Wilder et al., 2007) during transitions. These studies showed that advance notice in the form of vocal statement or question did not reduce challenging behaviors during transitions (McCord et al., 2001). One possible explanation for the discrepancy in effectiveness is that advance notice will likely not work if the challenging behavior is not controlled by unpredictability of transitions (Brewer et al., 2014; Castillo et al., 2018).

Another account for why transitions may evoke challenging behavior is that the transition involves negative incentive shifts (Brewer et al., 2014; Castillo et al., 2018). Negative incentive shifts occur when the density of reinforcement decreases as an individual transitions from a context associated with rich reinforcement to a context associated with relatively lean reinforcement. For example, challenging behavior may occur when an individual transitions from a highly preferred activity to a relatively less preferred activity. Negative incentive shifts have been demonstrated and generalized across different topographies of transition-related challenging behavior (e.g., aggression), different schedules of reinforcement (e.g., fixed-interval), and species (e.g., humans, rats, pigeons; Perone & Courtney, 1992; Pitts et al., 2019; Toegel & Perone, 2022; Wade-Galuska et al., 2005; Williams et al., 2011, 2019). These studies showed that individuals tend to avoid or escape the aversive aspects of the rich-lean transition. In these cases, simply signaling transitions may not be effective at reducing challenging behavior (Brewer et al., 2014; Castillo et al., 2018). To address situations where the transition involves negative incentive shifts, researchers have used other procedures that do not involve a signaling stimulus and incorporated interventions directed at the consequences (e.g., differential reinforcement, extinction, and blocking; Dowdy & Tincani, 2020; McCord et al., 2001; Waters et al., 2009).

Understanding the controlling variables (i.e., unpredictability and negative incentive shifts) that contribute to transition-related challenging behavior may be critical to determining effective interventions. Several reviews have been conducted to examine the interventions for transition-related challenging behaviors (Banda & Grimmett, 2008; Koyama & Wang, 2011; Lequia et al., 2012; Sterling-Turner & Jordan, 2007). These reviews, however, either focused on the use of activity schedules (Banda & Grimmett, 2008; Koyama & Wang, 2011; Lequia et al., 2012) or provided a brief review of studies primarily incorporating interventions directed at antecedents (e.g., verbal and visual cues) in individuals with autism. Little research has examined the controlling variables of transition-related challenging behavior and quantified and compared the effectiveness of the interventions.

Therefore, the purpose of the current review was to analyze interventions targeting transition-related challenging behavior and to compare the effectiveness of the different types of procedures in studies published in behavior-analytic journals. Specifically, this review focused on *transition from one activity to another* rather than contextual or developmental transitions. Based on the hypothesized controlling variables of transition-related challenging behavior (i.e., unpredictability and negative incentive shifts; Brewer et al., 2014; Castillo et al., 2018), we categorized the interventions of the identified experiments as including a signaling stimulus or utilizing other types of interventions to reduce transition-related challenging behavior. A targeted research synthesis was conducted to answer the following research question: What type of signaling stimulus and other interventions were used to address transition-related challenging behavior? A multilevel meta-analysis was conducted to answer the following research questions: First, what are the overall effects of interventions on challenging behavior for individuals with disabilities? Second, is there intervention heterogeneity between participants and between studies? If there is heterogeneity in intervention effectiveness, then the following research question will be further investigated: Does the type of intervention (i.e., interventions with or without signaling stimulus) explain differences in intervention effectiveness? In other words, is intervention type a statistically significant moderator?

## Method

### Article Search

**Figure 1.**
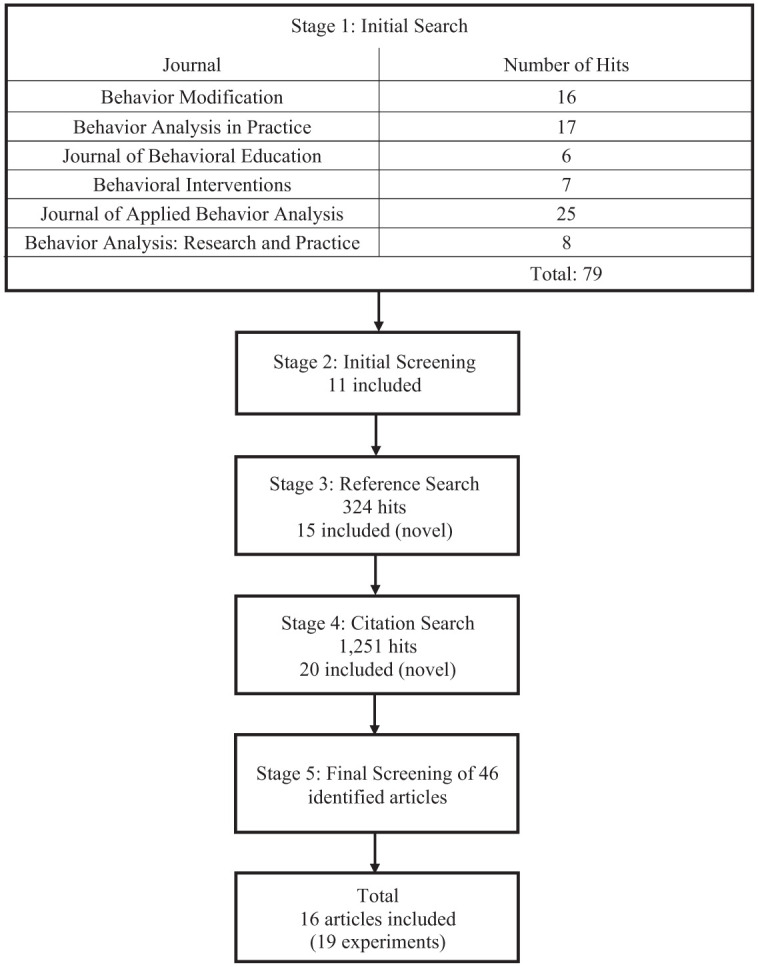
Schematic overview of article search and screening.

The article search was conducted in five stages—initial search, initial screening, reference search, citation search, and final screening. Figure 1 shows a schematic overview of the article search and screening with the number of articles identified and included in each stage. Given the goal of summarizing the state of behavior-analytic research specifically addressing transition-related challenging behavior, this review was intentionally designed as a targeted research synthesis rather than a comprehensive systematic review spanning all disciplines. Articles were analyzed from six specific behavior-analytic journals (*Behavior Modification*, *Behavior Analysis in Practice*, *Journal of Behavioral Education*, *Behavioral Interventions*, *Journal of Applied Behavior Analysis*, and *Behavior Analysis: Research and Practice*) to best reflect behavior-analytic practice related to transition-related challenging behaviors. These journals were specifically selected because they represent the primary outlets for applied behavior-analytic research and are among the most frequently referenced, peer-reviewed sources publishing intervention studies with single-case experimental designs relevant to individuals with developmental disabilities. Although this approach necessarily excluded journals outside of behavior analysis, it was intended to ensure that all included studies reflected the conceptual and methodological rigor characteristic of applied behavior-analytic research. The implications of limiting the search to these journals are acknowledged as a limitation in the “Discussion” section.

#### Inclusion and Exclusion Criteria

The articles identified through the data search procedure were evaluated for inclusion in the review. To be included, an article needed to (a) use the term *transition* or describe a change from one activity to another, (b) describe that an occurrence of challenging behavior was related to activity-related transitions, (c) include participants consisting of individuals with developmental disabilities, (d) be an experimental study (i.e., an independent variable was actively manipulated to observe its effect on the dependent variable), (e) be peer-reviewed, and (f) be written in English. Only peer-reviewed articles were included in this review, and gray literature (e.g., dissertations, conference proceedings, unpublished reports) was excluded. This decision was made to ensure that all included studies underwent formal peer review, thereby maintaining methodological rigor and consistency in the quality of the data analyzed.

An article was excluded if (a) the term *transition* related to contextual or developmental transitions, rather than from one activity to another, (b) the activity-related transitions were not associated with challenging behavior, or (c) it was a systematic review, meta-analysis, or conceptual paper.

#### Stage One: Keyword Search

An initial search was conducted through the PsycINFO database using the keyword “transition” in the abstract section. The search was conducted in July 2023. This search was conducted individually for six specific behavior analytic journals mentioned above. The initial search yielded a total of 79 results across the six journals.

#### Stage Two: Initial Screening

The articles were then further analyzed based on the inclusion criteria. If there was any mention of challenging behavior (e.g., aggression, noncompliance) in the abstract, the entire paper was further analyzed to assess whether the challenging behavior was related to activity transitions. Based on these inclusion criteria, 11 articles were selected for Stage Three.

#### Stage Three: Reference Search

This stage was the reference search, which was conducted for the articles that met the inclusion criteria in Stage One and Two (*n* = 11). The references from those articles were collated to produce 324 articles, and these included several duplicates. These identified articles were then analyzed based on the inclusion criteria mentioned in Stage One and Two. Only articles from the specified six journals were included. The reference search resulted in 15 new articles.

#### Stage Four: Citation Search and Screening

A citation search was conducted for the articles that were retrieved from Stage One, Two, and Three (*n* = 26) using the Google Scholar database. The search was conducted by exporting the references from the “Cited by” link and selecting to display articles through the July of 2023. The citation search yielded 1,251 search results. As in prior stages, only articles published in the six predetermined behavior-analytic journals were considered for inclusion. Each article identified through the citation search was analyzed to ensure that it met the same inclusion criteria described in Stage Two. A new citation search was conducted for each newly identified article during this stage. After completing several rounds of citation searches and analyzing them based on the inclusion criteria, a total of 20 new articles were identified.

#### Stage Five: Final Screening

After completing the first four stages, a preliminary list of 46 articles was collated. These articles were then further analyzed based on their entire body of text. A total of 16 articles met these criteria. There were 19 experiments within the 16 articles. Thus, a total of 19 unique experiments were analyzed for the current review.

#### Article Search Interobserver Agreement

Interobserver agreement was calculated by an independent researcher for Stage One, Two, Three, and Four of the article search process. For Stage One, the independent researcher first replicated the keyword search for all six journals. Agreement was 100% in terms of the total articles identified. For Stage Two, the reviewer analyzed a randomized list of 100% of the search results (*n* = 79) using the inclusion criteria specified in Stage One and Two and agreement was 100%. For both Stage Three and Stage Four, the reviewer was provided with a randomized list of 33.00% of the articles (*N* = 108 and 417, respectively) and was asked to analyze and apply the specified inclusion criteria to determine articles which were relevant for the review. Agreement for Stage Three and Four was 99.07% and 98.56%, respectively.

### Final Count of Experiments for Analysis

For the purpose of this review, an experiment was defined as a distinct evaluation of an intervention using an experimental design (e.g., single-case) that included an independent manipulation of variables and corresponding outcome data. Some articles contained multiple experiments that met the inclusion criteria, whereas others included a single relevant experiment. A total of 19 experiments were identified across the 16 included articles during the search procedure.

However, some articles included more than one distinct experimental comparison relevant to the current review (e.g., McCord et al., 2001; Waters et al., 2009). In these cases, each independent manipulation of the intervention variable was coded as a separate experiment when it was associated with its own baseline and intervention phases and evaluated distinct procedures or intervention components. This decision allowed for a more refined research synthesis and meta-analysis, capturing all relevant evaluations of interventions for transition-related challenging behavior rather than collapsing multiple experimental manipulations into a single data point. As a result, for the targeted research synthesis, the total number increased to 24 because certain experiments contained multiple independent comparisons (e.g., separate participants or conditions analyzed as unique data series). For the meta-analysis, 22 experiments were analyzed after the removal of an outlier and one group design study.

### Data Analysis for Targeted Research Synthesis

For each of the 24 experiments, data were extracted on the (a) general characteristics of the literature, which included information on citation, participants, setting, and pre-intervention functional analysis and (b) transition-related challenging behavior interventions, which included information on the independent variables(s) and controlling variable(s).

#### General Characteristics of the Literature

##### Citation

Article information such as authors, name of paper, year of publication, journal name, issue number, and page number were collected.

##### Participants

Participant information regarding the number of participants, participants’ age, gender, diagnoses, and demographics was extracted.

##### Setting

The location where the experiment was conducted was extracted.

##### Pre-Intervention Functional Analysis

If an experiment incorporated a functional analysis prior to implementation of the intervention, the results of functional analyses were extracted. The graphical representation of the functional analysis and the results described by the researchers in the article were used to note the function of the target behavior.

#### Transition-Related Challenging Behavior Interventions

##### Independent Variables(s)

Procedural information of the independent variable was extracted. Independent variables were categorized according to whether they included a signaling stimulus or utilized other procedures. If a signaling stimulus was used, further description of the intervention and the type of signaling stimulus were noted. If other procedures were used (i.e., no signaling stimulus), further descriptions of the intervention and the underlying principle of the procedure were noted. When an experiment was comparative (i.e., comparing the use of signaling stimulus to another intervention), each independent variable was individually examined. Because two independent variables were coded from Mace et al. (1998), and three independent variables were coded for McCord et al. (2001) and Waters et al. (2009), the total number of experiments analyzed increased to 24.

##### Controlling Variable(s)

Once the independent variables were categorized based on the use of signaling stimulus, the review further coded whether each procedure accounted for unpredictability and/or negative incentive shifts based on the hypothesized controlling variables of transition-related challenging behavior (Brewer et al., 2014; Castillo et al., 2018). Accounting for unpredictability meant that the procedure aimed at reducing unpredictability by signaling when the current activity will end and what the next activity will be. Accounting for negative incentive shifts meant that the procedure aimed at mitigating the effects of the change in the density of reinforcement as an individual transitions from a context associated with rich reinforcement to a context associated with relatively lean reinforcement.

#### Data Analysis Interobserver Agreement

A second reviewer independently analyzed a randomized list of 25% of the experiments (*n* = 6) selected by the first author. Agreement was calculated using point-by-point agreement and the equation agreements divided by agreements plus disagreements multiplied by 100. The reviewer analyzed the articles to assess whether the experiments fit the inclusion criteria, whether functional analysis was conducted and if so, what the results were, and whether the intervention incorporated a signaling stimulus. Agreement was 100%.

### Data Analysis for Meta-Analysis

#### Descriptive Statistics

To prepare for the three-level meta-analysis, we explored the number of observations within baseline versus intervention phase, the number of participants within studies, and the total number of studies available for quantitative integration. We investigated the descriptive statistics of the standardized baseline and intervention data to identify potential outliers and influencing points. Outcome data were extracted from the graphical representation of the results of each experiment using PlotDigitizer (https://plotdigitizer.com/). Note that only data from the first baseline and intervention phases was used. Three participants were removed as outliers (more detail available in “Results” section). The data used for this analysis are openly available in the Open Science Framework (OSF) at https://osf.io/4q8bu.

#### Inferential Statistics

The multilevel meta-analysis framework, which considers the nested data structure, was used to combine SCED data across participants and across experiments (Moeyaert et al., 2014). Moreover, multilevel analysis captures the variability in intervention effectiveness between experiments and between participants and allows modeling moderators to explain variability (Moeyaert, Xue, & Yang, 2023). The statistical appropriateness of the multilevel meta-analytic framework for single-case meta-analysis has been validated through methodological work (i.e., for an overview, see Moeyaert, Dehghan-Chaleshtori et al., 2023) and indicated that reliable and valid estimates of fixed effects (i.e., intervention effectiveness) can be obtained with sufficient statistical power with as few as 10 experiments (Moeyaert et al., 2013). Recent methodological work further evidenced the appropriateness of the multilevel modeling framework to include participant and experiment moderators to explain intervention heterogeneity (Moeyaert, Xue, & Yang, 2023). Consequently, in this study, a general multilevel meta-analysis was run to investigate the overall effectiveness of the intervention and a subsequent multilevel meta-regression mode was run to investigate the influence of the moderator intervention type on intervention effectiveness.

The statistical model used to estimate the overall intervention effect size across experiments is displayed in equation (1).

(1) Yijk=γ000+v00k+u0jk+(γ100+v10k+u1jk)Dijk+eijkwitheijk=ρ1e(t−1)jk+εijkwithεtjkN~iid(0,σεjk2),[u0jku1jk]∼N(0,Σu),and[v00kv10k]∼N(0,Σv)

In this equation, the outcome is referred to as $Y_{ijk}$ and reflects the outcome on observation occasion *i* (*i* = 0, 1,. . .*I*), nested within participant *j* (*j* = 1, 2,. . .*J*), which in turn is nested within experiment *k* (*k* = 1, 2, 3, 4, . . .*K*). $D_{ijk}$ represents the experiment phase and is a dummy coded variable with zero for the baseline phase and one for the intervention phase. The expected baseline level across participants and experiments is represented by $\gamma_{000}$ , while $\gamma_{100}$ represents the expected change in outcome level between the baseline phase and the intervention phase. The deviation of experiment *k* from the overall expected baseline is captured by $v_{00k}$ . $v_{10k}$ stands for the deviation of experiment *k* from the overall expected change in level. The values $u_{0jk}$ and $u_{1jk}$ , represent the deviations of individual participants *j* from experiment *k* from the overall expected baseline and overall expected change from baseline to treatment phase. *e_ijk_* is the residual term and lag−1 autocorrelation (i.e., $\rho_{1}$ ) is assumed for these residuals taking into account that repeated measures are correlated (see Moeyaert et al., 2014 for more information and further interpretations). In order to combine the raw outcomes (i.e., *$Y_{ijk}$ *) across studies, the outcomes were standardized following the procedure outlined by Van den Noortgate and Onghena (2007) and empirically validated by Moeyaert et al. (2013). Equation (1) was further expanded by including the moderator intervention type. For an in-depth discussion of incorporating moderators in the multilevel modeling framework (i.e., meta-regression), see Moeyaert, Xue, and Yang (2023). The statistical software R (Version 4.5.0) was used to run all analyses.

## Results

The following section describes the results of the findings by discussing the general characteristics of the literature, targeted research synthesis research question related to the characteristics of transition-related challenging behavior interventions, and multilevel meta-analysis research question related to the effectiveness of the interventions.

### General Characteristics of Literature

**Figure 2.**
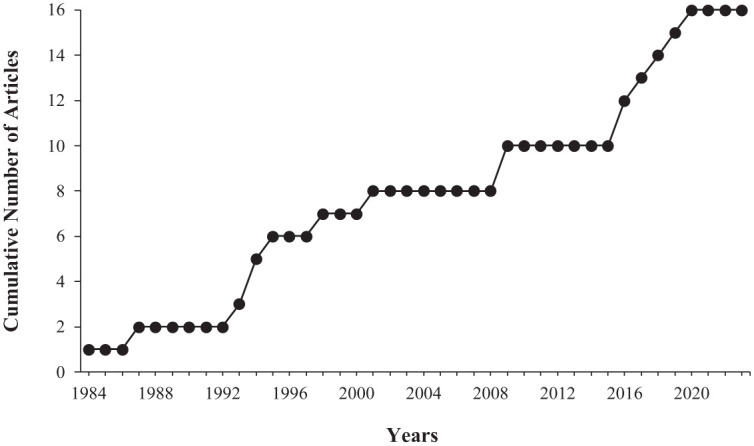
Cumulative record graph of the publication years of included articles.

The *Journal of Applied Behavior Analysis* published the majority of articles included in this search (see Table 1). There was a similar ratio of articles that were published in the first two decades (1980–1999, *n* = 7 articles), as compared to the last two decades (2000–2023, *n* = 9 articles; see Figure 2). Tables 2 and 3 display the number of participants, age, diagnoses, and setting information from each included experiment. The majority of the experiments included participants who were under the age of 18 (*n* = 20 out of 24 experiments; 83.33%), with the youngest being 3 years old and the oldest being 38. Male participants were included in most experiments (*n* = 21 out of 23^1^ experiments; 91.30%), and most of the experiments included participants with an Autism Spectrum Disorder (ASD) diagnosis. The most common diagnosis was Autism Spectrum Disorder (ASD) (15 out of 24 experiments; 62.50%), and only one experiment (4.17%) reported the demographics information on the participants (Iadarola et al., 2018).

**Table 1.** Publishing Journals.

Journal	# Articles (# experiments)
*Journal of Applied Behavior Analysis*	10 (11)
*Behavior Analysis in Practice*	3 (3)
*Behavior Modification*	2 (3)
*Journal of Behavioral Education*	1 (2)

**Table 2.** List of Signaling Stimuli in Included Experiments.

	Signaling stimulus		Participant and setting information				Controlling variable	
Article	Types	Description	Number of participants (M/F)	Age range	Diagnosis	Setting	Unpredictability	Negative incentive shifts
Aspiranti et al. (2018)	Visual and verbal	Tri-colored poster board with corresponding rule presented 2 min and 30 s prior to transition	14/7	5–11	ASD	School	X	
Cale et al. (2009), Experiment 1	Visual and verbal	Visual schedule of the day’s activities highlighting the transitions; verbal warning prior to transition; a cue card to prompt the individual to ask the teacher if they have missed anything (in case they are joining an activity which has already begun)	1/2	5–8	Aspergers syndrome, pervasive developmental disorder	School	X	X
Cale et al. (2009), Experiment 2	Visual and verbal	Countdown cards visually and vocally presented prior to transition	3/0	6–7	ASD	School	X	
Flannery and Horner (1994), Experiment 1	Gestural and verbal	Vocal description and modeling of task sequences	1/0	14	ASD	School, Church	X	
Flannery and Horner (1994), Experiment 2	Visual and verbal	Printed list of activities for the day presented with verbal prompt to reply with the upcoming activity	1/0	17	ASD	School	X	
Huffman et al. (2016)	Visual and verbal	Task analysis with visual prompts of behaviors during transitions (e.g., cleaning up) presented with verbal prompts	1/0	6	Down syndrome	School	X	X
Iadarola et al. (2018)	Verbal	Vocal warning for transition and description of instructions and behavioral expectations prior to transition	131/19	K–6th Grade	ASD	School	X	
Mace et al. (1998)	Visual and verbal	Picture prompt and warning statement once every 30 s, for a total of four presentations within 2 min prior to transition	0/1	7	ASD, intellectual disability	School	X	
McCord et al. (2001)	Verbal	Vocal presentation of the next scheduled activity 2 min prior to transition	2/0	27–38	Intellectual disability, visual impairment	Home	X	
Sainato et al. (1987)	Visual	Visual presentation of a bell where individual is asked to go to a different area and ring the bell; visual prompt of a smiley face where a peer would assist the individual with the transition; visual prompt of a green stop light where individuals would go to a designated area	3/0	3–4	ASD	School	X	
Tustin (1995)	Visual and verbal	Presentation of alternative materials and asking if individual would like to start the upcoming activity 2 min prior to transition	1/0	28	ASD, intellectual disability	Work	X	
Vasquez et al. (2017)	Visual and verbal	Vocal warning in the beginning of transition and presentation of alternative materials for upcoming activity 2 min prior to transition	1/0	7	ASD	Home	X	
Waters et al. (2009)	Visual	Visual schedule representing current and upcoming activities	2/0	6	ASD	School	X	
Waters et al. (2009)	Visual and verbal	Vocal warning and visual schedule representing current and upcoming activities	2/0	6	ASD	School	X	X

*Note.* M = male; F = female; ASD = autism spectrum disorder; K = kindergarten.

**Table 3.** Interventions Following Other Principles in Included Experiments.

	Other intervention		Participant and setting information				Controlling variable	
Article	Description	Principle	Number of participants (M/F)	Age	Diagnosis	Setting	Unpredictability	Negative incentive shifts
Connell et al. (1993).	Self-assessment with corrective feedback or praise on completing tasks; recruiting praise in an appropriate manner for completing transition steps successfully	Self-management	3/1	4	Language and cognitive delays	School		X
Dowdy and Tincani (2020)	Differential reinforcement of alternative behavior without extinction	Contingency-based positive reinforcement	2/0	10–17	ASD, ADHD, Marfan’s syndrome, DiGeorge syndrome, intermittent explosive disorder, pica, intellectual disability	Residential treatment facility		X
Jessel et al. (2016), Experiment 2	Utilizing a “mystery” box where the activity might be more preferred or less preferred on a random basis	Probabilistic availability of relatively preferred activities/items	3/0	3–6	ASD	University clinic		X
Mace et al (1998)	Extinction and noncontingent reinforcement for 15 to 20 s on a fixed-time 60-s schedule	Extinction; noncontingent reinforcement	0/1	7	ASD, intellectual disability	School		X
McCord et al. (2001)	Differential reinforcement of alternative behavior	Contingency-based positive reinforcement	2/0	27–38	Intellectual disability, visual impairment	Home		X
McCord et al. (2001)	Differential reinforcement of alternative behavior with extinction and response blocking	Contingency-based positive reinforcement; extinction; negative punishment	2/0	27–38	Intellectual disability, visual impairment	Home		X
Repp and Karsh (1994)	Differential reinforcement of alternative behavior with extinction; differential reinforcement of alternative behavior; increasing the number of opportunities to respond and to engage socially	Contingency-based positive reinforcement; extinction; motivating operations	2/0	7–9	Intellectual disability, down syndrome, cerebral palsy	School		X
Smith and Fowler (1984), Experiment 1	Peer-monitored token system	Contingency-based positive reinforcement; group contingency	Unclear (3 in total)	5–7	Language and developmental delays	School		X
Smith and Fowler (1984), Experiment 2	Peer-monitored token system with and without corrective teacher feedback	Contingency-based positive reinforcement; group contingency	2/1	5–7	Language and developmental delays	School		X
Waters et al. (2009)	Extinction and differential reinforcement of other behavior	Contingency-based positive reinforcement extinction	2/0	6	ASD	School		X

*Note.* M = male; F = female; ASD = autism spectrum disorder; K = kindergarten.

Experiments were also analyzed to assess whether a functional analysis was conducted prior to the implementation of the intervention. Results reflected that nine out of 19 experiments (47.36%) performed an experimental functional analysis (Iwata et al., 1994) of the transition-related challenging behavior (listed in Table 4). The remaining experiments included indirect assessments or reports from parents and/or teachers regarding the challenging behavior and the context in which they occurred. For example, Cale et al. (2009) prompted parents of the participants to complete the Contextual Assessment Inventory (CAI), which was then confirmed by the teachers who worked with the participants. The purpose of CAI was to help identify the contexts, such as discriminative stimuli or setting events, that evoked challenging behavior. A summarized list of the results of the functional analyses is presented in Table 4. For participants who took part in functional analyses, it was found that the primary function of the behavior was escape (negative reinforcement; *n* = 8 participants) followed by access to tangibles or activity (positive reinforcement; *n* = 7 participants) and attention (social positive reinforcement; *n* = 3 participants).

**Table 4.** Results of Functional Analysis from Included Experiments.

Article	Functional analysis results
Dowdy and Tincani (2020)	Escape and access to a preferred activity (2 participants)
Flannery and Horner (1994), Experiment 1	Escape (1 participant)
Flannery and Horner (1994), Experiment 2	Escape (1 participant)
Huffman et al. (2016)	Attention (1 participant)
Mace et al. (1998)	Escape and access to restricted tangibles (1 participant)
McCord et al. (2001)	Avoidance (1 participant), avoidance and escape (1 participant)
Repp and Karsh (1994)	Attention (2 participants)
Vasquez et al. (2017)	Access to prior activity (1 participant)
Waters et al. (2009)	Escape and access to a preferred activity (2 participants)

### Targeted Research Synthesis Research Question: What Type of Signaling Stimulus and Other Interventions Were Used to Address Transition-Related Challenging Behaviors?

Independent variables from the experiments were categorized into (a) interventions including a signaling stimulus and (b) other types of interventions with no signaling stimulus. Interventions that included a signaling stimulus referred to procedures that presented the participant with a stimulus to signal transition before a transition occurred (see Table 2 for the list and description of the signaling stimuli used in each experiment). Other interventions included procedures that did not include a signaling stimulus and rather employed other principles (see Table 3 for the list of interventions and principles for each experiment). Fourteen experiments included a signaling stimulus and 10 experiments included interventions using other principles.^2^

Across the experiments incorporating a signaling stimulus, verbal signaling stimulus (e.g., vocal warnings, rules, description, ask; *n* = 12) was most widely used, followed by visual (e.g., visual schedule, alternative materials, picture prompts, countdown cards, visual presentation of rules; *n* = 11) and gestural (e.g., modeling; *n* = 1) signaling stimulus. Commonly used stimuli included vocal warnings, visual schedules, and picture prompts. Most of the visual stimuli were paired with vocal verbal stimuli prior to the transition (e.g., visual schedule and warning statement; Aspiranti et al., 2018; Cale et al., 2009; Experiment 2 of Flannery & Horner, 1994; Mace et al., 1998; Tustin, 1995; Vasquez et al., 2017; Waters et al., 2009). Some studies reported the timing of the signaling stimulus, and when they did, 2 min warning prior to the transition was reported most frequently (*n* = 4; Mace et al., 1998; McCord et al., 2001; Tustin, 1995; Vasquez et al., 2017).

Other interventions that were included in this review did not employ a signaling stimulus but rather used other principles as the basis of the intervention. These interventions consisted of consequence-based procedures, which included principles of contingency-based positive reinforcement (*n* = 7), extinction (*n* = 4), group contingency (*n* = 2), noncontingent reinforcement (*n* = 1), motivating operations (*n* = 1), and probabilistic availability of relatively preferences activities/items (*n* = 1). Commonly used procedures included differential reinforcement with extinction (McCord et al., 2001, Repp & Karsh, 1994; Waters et al., 2009).

The experiments were further coded for whether the intervention accounted for the possible controlling variables (i.e., unpredictability and negative incentive shifts; see Tables 2 and 3 for detailed information). All 14 experiments using signaling stimulus as interventions accounted for unpredictability by signaling when the current activity will end and what the next activity will be. Three experiments also accounted for negative incentive shifts (i.e., used procedures mitigating the effects of the change in density of reinforcement) by incorporating a preferred peer during transitions (Experiment 1 of Cale et al., 2009), access to preferred items for completing a task analysis (Huffman et al., 2016), and providing reinforcement for the absence of challenging behavior (i.e., differential reinforcement of other behavior [DRO]; Waters et al., 2009). All 10 experiments using other types of interventions accounted for negative incentive shifts.

### Multilevel Meta-Analysis Research Questions: What Are the Overall Effects of Interventions on Challenging Behavior for Individuals with Disabilities? And Is There Intervention Heterogeneity Between Participants and Between Studies?

#### Descriptive Statistics

Data from 22 experiments were available for quantitative integration. The number of experiments was 22 because, out of the 24 total experiments, a group design study (Iadarola et al., 2018) was removed, and all participants from Waters et al. (2009) were removed as outliers, eliminating the experiment itself from the analysis. Across these 22 experiments, data from 62 participants were available with a median of three participants per studies (*M* = 2.70) having a range from 1 to 6. Next, across all participants and all experiments, a total of 1,672 observations were obtained. The median number of total observations per participant equaled 19.5 (*M* = 26.97) with a range from 6 observations to 111 observations. We also investigated the average number of observations during the baseline and during the intervention phase separately. The median number of observations obtained during the baseline phase equaled 6 (*M* = 10.05, range = 3–58) whereas the median number of observations during the intervention phase equaled 13 (*M* = 16.91, with range = 3–54).

**Figure 3.**
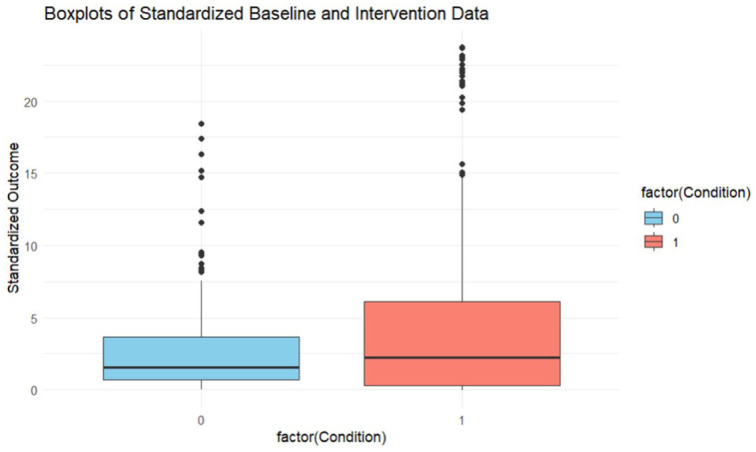
Distribution of standardized outcome scores. *Note.* 0 = Baseline; 1 = Intervention.

To combine outcomes across cases and across experiments, standardization was needed. This was accomplished by dividing the $Y_{ijk}$ outcomes by the residual standard deviation as outlined by Van den Noortgate and Onghena (2007) and empirically validated by Moeyaert et al. (2013). Similar to group design studies, it is impossible to standardize if there is zero variability in baseline and intervention data (i.e., a zero in the denominator is not possible). Therefore, data from three experiment participants needed to be removed, which still resulted in data from 59 participants across 22 experiments available for quantitative synthesis. The boxplot in Figure 3 provides the distribution of the estimated standardized outcomes for the baseline phase and intervention phase separately. The average standardized outcome during the baseline was 2.72 (*SD* = 3.12, *Mdn* = 1.56) and ranged from 0 to 18.42. The skewness was 2.12 which only slightly exceeds the 2.00 cut-off score (i.e., indicating that there might be positive outliers skewing the distribution to the right). The average standardized outcome during the intervention was 3.66 (*SD* = 4.33, *Mdn* = 2.21) and ranged from −0.03 to 23.77. The skewness was 1.96. Because the skewness of data during the baseline and intervention were either lower then or slightly exceeding the 2.00 cut-off score, all standardized outcome scores were retained.

#### Inferential Statistics for Main Analysis: Three-Level Hierarchical Linear Model

The overall average weighted intervention effect size (standardized) across all 59 participants and 22 experiments equaled −1.07 [*SE* = 0.98, 95% CI [−3.01, 0.86], *t* (1,567) = −1.09, *p* = .275]. This indicated there was a decrease of 1.07 standardized units in the intervention phase outcomes compared to the baseline phase. There was a large amount of both between-case and between-experiment variability in intervention effectiveness (i.e., $\hat{\sigma_{v_{1}}^{2}}$ = 12.43 and $\hat{\sigma_{u_{1}}^{2}}$ = 19.61). The estimated autocorrelation was 0.247 providing evidence that the repeated measures were correlated. Altogether, this indicated that across all studies, the intervention resulted in a decrease in outcome scores during the intervention phase, even though this decrease was not statistically significant. However, there was a lot of variability in intervention effectiveness, indicating that some study participants might have benefited more from the intervention than others. Therefore, in the next step, we added the moderator intervention type that was hypothesized to explain intervention heterogeneity.

#### Inferential Statistics for Moderator Analysis

Data for 59 participants across 22 experiments were available for analysis. The estimated standardized intervention effect for participants with Intervention.Type = 0 (i.e., interventions using a signaling stimulus) equaled 0.58 [*SE* = 1.86, 95% CI [−1.92, 3.08], *t* (1,566) = 0.46, *p* = .649], which indicated that the overall average weighted standardized outcome during the intervention phase was slightly higher compared to the baseline phase. In contrast, the estimated standardized intervention effect for participants with Intervention.Type = 1 (i.e., interventions without a signaling stimulus) equaled −3.32 [*SE* = 1.86, 95% CI [−6.96, 0.32], *t* (1,566) = −1.79, *p* = .0741]. This provides evidence that using interventions without signaling stimuli resulted in a large decrease in outcome during the intervention phase compared to the baseline phase. This effect was not statistically significant most likely because of lack of power. Notwithstanding, the *p*-value was approaching statistical significance at the significance level of .05 for two-tailed testing (*p* = .0741). For one-directional testing, the moderator effect was statistically significant.

## Discussion

The purpose of this targeted research synthesis and meta-analysis was to synthesize and compare the existing interventions published in behavior-analytic journals aimed at reducing transition-related challenging behavior displayed by individuals with IDD. Research has hypothesized two potential controlling variables for why transition-related challenging behavior occurs: unpredictability or negative incentive shifts (Brewer et al., 2014; Castillo et al., 2018). Adopting these hypotheses as a foundational basis, we categorized the interventions of the identified experiments as including a signaling stimulus or utilizing other types of interventions to reduce transition-related challenging behavior. A multilevel meta-analysis was utilized to analyze the overall intervention effectiveness, intervention heterogeneity, and the moderating role of intervention type. In the following sections, we provide the two main findings, implications for practice and research, and limitations of the current review.

### Intervention Effectiveness and Heterogeneity

In line with previous literature, our findings indicate that a variety of procedures have been used to address transition-related challenging behaviors (Luczynski & Rodriguez, 2015; Mitteer et al., 2023). The multilevel meta-analysis indicated that, on average, interventions in general led to a reduction in transition-related challenging behavior. Although this decrease was not statistically significant, there was substantial variability both between participants and across experiments. This variability suggests that, while interventions in general may help decrease challenging behavior, the success is not uniform and depends on other individual and contextual factors.

When examining intervention type as a moderator, consequence-based interventions (i.e., no signaling stimulus) such as differential reinforcement, extinction, noncontingent reinforcement showed larger reductions in challenging behavior compared to interventions using signaling stimuli alone. Specifically, the estimated standardized intervention effect for consequence-based procedures approached statistical significance, suggesting a greater decrease in challenging behavior relative to baseline. In contrast, signaling stimulus interventions showed smaller and more variable effects.

The differential outcomes observed across intervention types likely reflect underlying behavioral mechanisms and contextual variables that influence treatment effectiveness. Interventions incorporating signaling stimuli (e.g., advance notice, visual schedules) are designed primarily to reduce unpredictability by establishing clear stimulus control over transitions. However, these procedures do not directly alter the reinforcement contingencies maintaining challenging behavior. When transition-related behavior is maintained by negative reinforcement (i.e., escape from task demands or transitions associated with leaner reinforcement), simply signaling the upcoming change may not reduce the aversive value of that transition. In contrast, consequence-based procedures such as differential reinforcement, extinction, or noncontingent reinforcement directly manipulate the contingencies that maintain challenging behavior, which may explain their relatively larger effect sizes in the current meta-analysis.

In addition, contextual variables such as the type of transition (e.g., preferred-to-nonpreferred activity), the presence of competing reinforcers, and individual sensitivity to negative incentive shifts may have moderated the effectiveness of interventions utilizing signaling stimuli. For example, advance notice might only be effective when unpredictability is the primary controlling variable. When the problem behavior is instead controlled by the relative loss of reinforcement density, signaling the transition could even exacerbate responding by extending exposure to the aversive condition. Collectively, the findings highlight the importance of matching intervention strategies to the specific controlling variables influencing transition-related behavior rather than applying signaling procedures universally.

It is worth noting that three articles conducted within-subject comparisons of various interventions with the same participants. Such comparisons provide valuable insight into the necessary procedures and components of an effective procedure for reducing transition-related challenging behavior and help address the “paucity of within-subject comparative analyses of treatment approaches for transition-related challenging behavior” (Mitteer et al., 2023, p. 268). Three articles in the current review (Mace et al., 1998; McCord et al., 2001; Waters et al., 2009) conducted a within-subject comparison of different procedures for reducing transition-related challenging behavior.

McCord and colleagues compared advance notice, differential reinforcement of alternative behavior (DRA), and DRA plus extinction plus blocking on the rate of self-injurious behavior (SIB) emitted by two adult males with intellectual disability during transitions. They found that advance notice and DRA alone were insufficient in reducing SIB in these individuals. In fact, advance notice alone yielded worsening results compared to baseline. Similarly, Waters and colleagues found that a visual schedule alone was ineffective at reducing transition-related challenging behavior (e.g., hitting and kicking others, throwing objects, falling to the floor) in two 6-year-old males diagnosed with autism. Only when extinction and DRO procedures were used (with and without visual schedules), the participants’ transition-related challenging behavior decreased. Further, Mace and colleagues compared the effects of extinction and noncontingent reinforcement (NCR) with and without a warning statement on a 7-year-old female diagnosed with autism and intellectual disability on SIB. Both conditions (i.e., extinction and NCR with and without a warning statement) were effective at reducing SIB compared to baseline, but adding the warning statement was more effective. Together, the three articles supported the findings from the multilevel meta-analysis that signaling stimulus alone may not be sufficient for decreasing transition-related challenging behavior and may need to be used in conjunction with other procedures such as DRA, DRO, NCR, extinction, and blocking.

### The Importance of Identifying Controlling Variables

Previous research has suggested two potential controlling variables of transition-related challenging behavior—unpredictability and negative incentive shifts (Brewer et al., 2014; Castillo et al., 2018). The findings in our review suggest that understanding the controlling variables that contribute to transition-related challenging behavior may be essential in determining the effectiveness of various interventions related to these behaviors. All 14 experiments incorporating signaling stimulus accounted for unpredictability, and all 10 experiments incorporating other types of interventions accounted for negative incentive shifts. There were three experiments (Cale et al., 2009; Huffman et al., 2016; Waters et al., 2009) that accounted for both unpredictability and negative incentive shifts.

Waters et al. (2009) provided valuable insights through their within-subject evaluation of three different procedures. Waters and colleagues performed a brief functional analysis and identified escape as one of the functions of the transition-related challenging behavior. Since individuals tend to avoid or escape the aversive nature of rich-to-lean reinforcement schedule transition (Perone & Courtney, 1992; Pitts et al., 2019; Toegel & Perone, 2022; Wade-Galuska et al., 2005; Williams et al., 2011, 2019), it is likely that negative incentive shift controlled the challenging behavior. It is possible that the use of extinction and DRA, both with and without visual schedule (Waters et al., 2009, respectively), was effective given that the procedure matched the underlying function of the challenging behavior. Similar findings were found in Mace et al. (1998) and McCord et al. (2001), which incorporated a functional analysis that identified the function of transition-related challenging behavior as avoidance and escape and used DRO, extinction, and NCR. Advance notice alone was not effective at reducing transition-related challenging behavior in these experiments, further highlighting that matching the intervention to the controlling variable improved effectiveness.

These findings highlight that functional analysis may be particularly useful in identifying the controlling variable of transition-related challenging behavior, especially in identifying whether the behavior is controlled by negative incentive shifts. Functional analysis (Iwata et al., 1994) has been replicated hundreds of times and numerous variations have surfaced since its inception (Beavers et al., 2013; Hanley et al., 2003; Melanson & Fahmie, 2023). These studies consistently demonstrated that considering the function of behavior prior to intervention selection increases the likelihood of the program’s success (Iwata et al., 1994; Vollmer & Iwata, 1992). If possible, experimentally identifying the function of the transition-related challenging behavior and selecting an intervention accordingly may lead to more effective results.

Although a functional analysis may be helpful in identifying negative incentive shifts as a controlling variable, a procedure to identify unpredictability as a controlling variable needs to be developed further. Flannery and Horner (1994) provide a potential method to identify the role of unpredictability. The authors compared random but signaled and random but not signalized conditions on the rate of transition-related challenging behavior. The high level of challenging behavior during random but not signalized condition and the low level of challenging behavior during random but signaled condition suggested unpredictability as a controlling variable. The limited success of using signaling stimulus in addressing transition-related challenging behavior as seen in the current review may be due to the arbitrary relationship between the reinforcer and target challenging behavior (Vollmer & Iwata, 1992). Future practitioners and researchers should consider using a similar approach to Flannery and Horner (1994) to isolate the effect of unpredictability as a controlling variable and identify procedural components necessary for the success of the program.

It is important to note that the analysis of the controlling variables in the current review is preliminary and suggests future directions for practice and research. We categorized each experiment’s controlling variable based on the *aim* of the interventions. Unfortunately, this does not directly inform us of the controlling variable of the behavior itself. For example, a clinician may develop an intervention that aims to reduce the effect of unpredictability by using a visual schedule, but this does not experimentally inform us of the controlling variable of the behavior itself. As a result, the visual schedule may be ineffective at addressing transition-related challenging behavior. Future researchers should determine systematic ways to identify the controlling variables and assess the effectiveness of various procedures.

### Implications for Future Practice and Research

The findings in this review have several implications for future practice and research, although these implications should be interpreted with caution given the limited and heterogeneous evidence base. First, when a practitioner is deciding on an intervention for transition-related challenging behavior, we strongly encourage them to determine the controlling variable of the transition-related challenging behavior as the first step. Conducting a functional analysis could help practitioners identify the variables maintaining transition-related challenging behavior and thereby guide intervention selection. In applied settings, identifying the controlling variable can be achieved through systematic observation and brief assessment procedures. For example, practitioners can conduct structured descriptive assessments or brief experimental analyses that manipulate the predictability of transitions (e.g., comparing conditions with and without advance notice) or the reinforcement context (e.g., preferred-to-nonpreferred activity shifts). Observing whether challenging behavior occurs primarily when transitions are unpredictable or when reinforcement density decreases can help determine whether unpredictability or negative incentive shifts are controlling the behavior. When formal functional analysis (Iwata et al., 1994) is not feasible, indirect tools such as transition-specific checklists or structured interviews with teachers and caregivers can provide preliminary hypotheses to guide intervention selection (see Melanson & Fahmie, 2023 for the latest review on functional analysis and variations in literature).

Our findings suggest that, if unpredictability of the following activity is causing the challenging behavior, a practitioner should consider using a signaling stimulus (e.g., advance notice, visual schedule). Flannery et al. (1995) identified certain strategies for using advance notice such as identifying the key variable of predictability, identifying the appropriate method in which predictability can be conveyed to the individual, and ensuring that the individual has consistent access to the information to make an individual’s environment more predictable. If the use of a signaling stimulus does not improve challenging behavior, the practitioner may want to consider using advance notice in conjunction with other types of procedures directed at the consequence (e.g., DRA, DRO, NCR, extinction, blocking).

If negative incentive shift is controlling the challenging behavior, a practitioner may want to use contingency-based positive reinforcement procedures such as DRA and DRO. These procedures should be carefully considered based on the result of the functional analysis. Previous literature has suggested that, if escape/avoidance is the function of the challenging behavior, a differential reinforcement procedure along with extinction (Piazza et al., 1996; Vollmer et al., 2022) may be effective at decreasing the challenging behavior. If attention is maintaining the challenging behavior, NCR may be effective as it reduces the target challenging behavior by minimizing the establishing operation (Carr et al., 2009; Piazza et al., 1997). Overall, these findings suggest that aligning interventions with the specific controlling variables of behavior could enhance effectiveness, but additional research is needed to substantiate these recommendations

It is important to note that there may be other controlling variables contributing to transition-related challenging behavior and that the list of interventions provided in the review is not exhaustive. Other interventions such as functional communication training (FCT; Boyle & Adamson, 2017) have been used to address transition-related challenging behaviors. FCT is commonly used for socially maintained challenging behavior and previous studies showed that it effectively decreased challenging behavior while increasing communication skills (Tiger et al., 2008). Other methods that have been suggested include probabilistic delays (Hanley et al., 2014), where a proportion of responses results in immediate reinforcement whereas others produce delays to reinforcement, and FCT with multiple and chained schedules of reinforcement (Greer et al., 2016). Limited research is available on these procedures addressing transition-related challenging behavior, and further research is warranted to investigate their effectiveness.

Second, evaluating the efficiency of the various procedures may be helpful for practitioners in determining an intervention targeting the reduction of transition-related challenging behavior. While the current review did not directly assess this variable, efficiency is valuable, especially in resource-intensive settings such as schools. Transition-related challenging behaviors often occur in educational settings, and previous studies have reported that teachers have difficulty regaining instructional control after a student engages in transition-related challenging behavior to and from the classroom (Colvin et al., 1997). Such difficulty may result in the loss of learning opportunities and distraction to peers. Thus, identifying strategies to minimize transition-related challenging behavior may be of interest to educators along with clinicians looking for procedures requiring minimal training and resources.

A few of the experiments included in the current review required more training and resources to carry out the procedures (e.g., Aspiranti et al., 2018; Iadarola et al., 2018). For example, Aspiranti et al. (2018) included a 60-minute workshop for teachers to learn about a classwide management system called the color wheel system and a systematic training system for data collectors. Iadarola et al. (2018) used an on-site hierarchical training model which included training staff coaches to carry out the training sessions that included “review of the manual, role-plays, in-vivo feedback, and . . . opportunities to observe supervisors implementing” (p. 136) the intervention. While these experiments provided novel ways to address transition-related challenging behavior, the amount of resources and training required prior to implementation of the procedures may make it difficult for educators to use the procedure effectively.

We encourage future researchers to empirically assess efficiency. Suggested methods include systematically measuring quantifiable aspects of the trainings and resources such as the amount of time it took to develop training materials, the number of individuals involved in the training, and the cost of implementation. We also encourage future researchers to evaluate efficiency along with effectiveness, as simply minimizing resources and training should not be the goal of any program.

Third, similar to efficiency, future researchers should evaluate the maintenance and generalization of the interventions addressing transition-related challenging behavior. Although the current review did not empirically analyze these variables, maintenance and generalization of skills should be considered, especially at the beginning of the planning stage, as transitions can occur in a variety of settings and between different activities or with different people who aid the individual with the transition. Further, ensuring maintenance and generalization will allow for the intervention to be gradually faded thus giving the individual an opportunity to function independently (Stokes & Baer, 1977). A few studies showed success in using schedule thinning (e.g., Kamlowsky et al., 2021; McCord et al., 2001) in addressing transition-related challenging behavior. For example, Kamlowsky et al. (2021) gradually thinned NCR with sustained success at reducing elopement. Another example is McCord et al. (2001) where the authors thinned the reinforcement schedule by requiring longer intervals of appropriate behavior. These studies provide potential methods for increasing maintenance and generalization for programs addressing transition-related challenging behavior.

### Limitations

One limitation of the current review was that we only searched a small pool of behavior-analytic journals. It is possible that there may have been additional behavior-analytic articles that were not identified through the process. Further, as transition-related challenging behavior is relevant to other disciplines such as education, developmental psychology, school psychology, occupational therapy, and speech-language pathology, there may have been relevant articles published in journals outside of behavior analysis. We encourage future researchers to incorporate broader search strategies that include a comprehensive list of journals to capture a wider range of relevant studies from multiple disciplines.

Second, the exclusion of gray literature in this review may have limited the comprehensiveness of the findings. Although restricting inclusion to peer-reviewed articles strengthened methodological rigor and ensured consistent reporting standards, it also potentially excluded relevant studies, particularly those with null results. Future reviews could broaden their scope to include gray literature sources to provide a more complete picture of intervention effectiveness and external validity across contexts.

Third, the articles included in the review were not analyzed for quality, and no formal risk of bias assessment was conducted. In other words, the methods and design of the experiments were not assessed with specific standards or quality indicators. This absence of a formal quality review limits the degree of confidence that can be placed in the aggregated findings, as the methodological rigor and internal validity of the included studies may have varied considerably. As the synthesis of research is unable to account for any issues that were built into the execution of the studies (Garg et al., 2008), utilizing a quality indicator tool can be beneficial in assessing the quality of the experiment and providing for a stronger evaluation. For example, What Works Clearinghouse (2020) provides standards for evaluating studies incorporating various designs such as single-case and group designs. These standards could be used to evaluate the quality and rigor of the published designs. Future studies should include such quality standards and formal risk of bias assessments to ensure a more robust and reliable evaluation of the findings.

Fourth, although the multilevel meta-analytic approach appropriately accounted for the nested data structure and variability across participants and experiments, statistical power to detect moderator effects may have been limited due to the relatively small number of experiments and participants. While the moderating effect of intervention type approached significance, the sample size may have constrained the ability to detect subtle differences between subgroups.

Fifth, the broad categorization of interventions into two groups (i.e., those incorporating a signaling stimulus and those using other procedures) presents an additional limitation. Although this classification was conceptually grounded in the hypothesized controlling variables of unpredictability and negative incentive shifts (Brewer et al., 2014; Castillo et al., 2018), it may have obscured meaningful variability among more specific intervention types. For example, procedures such as differential reinforcement, extinction, or noncontingent reinforcement may differ in their mechanisms and effectiveness, yet were analyzed within a single broad category. Future research should consider examining more fine-grained intervention categories or conducting component analyses to determine which procedural elements are most responsible for behavior change. Despite this limitation, the current categorization provided a useful first step in identifying patterns across the behavior-analytic literature and linking them to hypothesized underlying variables.

Sixth, a notable limitation concerns the limited use of experimental functional analyses across the included studies. Fewer than half of the experiments incorporated an empirical functional analysis to identify the maintaining variables of transition-related challenging behavior. This limitation is particularly important given the emphasis of the present review on matching interventions to controlling variables such as unpredictability and negative incentive shifts. Future research should incorporate experimental analyses explicitly designed to isolate the effects of unpredictability and reinforcement shifts to more rigorously link intervention mechanisms with behavioral function.

Finally, although we examined intervention outcomes during the baseline and treatment phases, we were unable to analyze long-term maintenance or generalization of effects due to limited follow-up data in the included studies. Given the importance of durable and contextually robust interventions, future research should include more systematic assessment of maintenance, generalization, and treatment fidelity.

## Conclusion

Our review compared different interventions designed to reduce transition-related challenging behavior published in behavior-analytic journals, based on the hypotheses underlying the controlling variables (i.e., unpredictability or shifts in negative incentive) that explain transition-related challenging behaviors. The findings suggest that identifying the controlling variables underlying transition-related behavior could help practitioners select more effective and individualized intervention strategies. Although the current evidence base remains limited and heterogeneous, this framework provides a useful starting point for conceptualizing why certain interventions are more effective under particular conditions. We hope that our findings will encourage practitioners in selecting appropriate approaches and inspire future research aimed at refining and extending behavior-analytic interventions for transitions.

## Notes

1. The total number of experiments is 23 here because one experiment (Smith & Fowler [1984), Experiment 1) did not specify the sex of the participants and thus was removed from calculation.

2. The total number of experiments in the results section starting from this point (*n* = 23) does not match the total number of experiments mentioned in the method section (*n* = 19) as comparison studies with multiple independent variables (i.e., comparing the effectiveness of a signaling stimulus procedure to other procedures) were analyzed at each independent variable level. Two independent variables were coded from Mace et al. (1998), and three independent variables were coded for McCord et al. (2001) and Waters et al. (2009).

## References

[bibr1-01454455261421144] *Aspiranti K. B. Bebech A. Ruffo B. Skinner C. H. (2018). Classroom management in self-contained classrooms for children with autism: Extending research on the color wheel system. Behavior Analysis in Practice, 12(1), 143–153. 10.1007/s40617-018-0264-6 30918777 PMC6411539

[bibr2-01454455261421144] Banda D. R. Grimmett E. (2008). Enhancing social and transition behaviors of persons with autism through activity schedules: A review. Education and Training in Developmental Disabilities, 43(3), 324–333.

[bibr3-01454455261421144] Beavers G. A. Iwata B. A. Lerman D. C. (2013). Thirty years of research on the functional analysis of problem behavior. Journal of Applied Behavior Analysis, 46(1), 1–21. 10.1002/jaba.30 24114081

[bibr4-01454455261421144] Brewer A. T. Strickland-Cohen K. Dotson W. Williams D. C. (2014). Advance notice for transition-related problem behavior: Practice guidelines. Behavior Analysis in Practice, 7(2), 117–125. 10.1007/s40617-014-0014-3 27540509 PMC4711751

[bibr5-01454455261421144] Boyle M. A. Adamson R. M. (2017). Systematic review of functional analysis and treatment of elopement (2000–2015). Behavior Analysis in Practice, 10(4), 375–385. 10.1007/s40617-017-0191-y 29214133 PMC5711741

[bibr6-01454455261421144] *Cale S. I. Carr E. G. Blakeley-Smith A. Owen-DeSchryver J. S. (2009). Context-based assessment and intervention for problem behavior in children with autism spectrum disorder. Behavior Modification, 33(6), 707–742. 10.1177/0145445509340775 19933441

[bibr7-01454455261421144] Carr J. E. Severtson J. M. Lepper T. L. (2009). Noncontingent reinforcement is an empirically supported treatment for problem behavior exhibited by individuals with developmental disabilities. Research in Developmental Disabilities, 30(1), 44–57. 10.1016/j.ridd.2008.03.002 18467073

[bibr8-01454455261421144] Castillo M. I. Clark D. R. Schaller E. A. Donaldson J. M. DeLeon I. G. Kahng S. (2018). Descriptive assessment of problem behavior during transitions of children with intellectual and developmental disabilities. Journal of Applied Behavior Analysis, 51(1), 99–117. 10.1002/jaba.430 29359370

[bibr9-01454455261421144] Colvin G. Sugai G. Good R. H. III Lee Y. Y. (1997). Using active supervision and pre-correction to improve transition behaviors in an elementary school. School Psychology Quarterly, 12(4), 344–363. 10.1037/h0088967

[bibr10-01454455261421144] *Connell M. C. Carta J. J. Baer D. M. (1993). Programming generalization of in-class transition skills: Teaching preschoolers with developmental delays to self-assess and recruit contingent teacher praise. Journal of Applied Behavior Analysis, 26(3), 345–352. 10.1901/jaba.1993.26-345 7691788 PMC1297757

[bibr11-01454455261421144] Cote C. A. Thompson R. H. McKerchar P. M . (2005). The effects of antecedent interventions and extinction on toddlers’ compliance during transitions. Journal of Applied Behavior Analysis, 38(2), 235–238. 10.1901/jaba.2005.143-04 16033169 PMC1226158

[bibr12-01454455261421144] *Dowdy A. Tincani M. (2020). Assessment and treatment of high-risk challenging behavior of adolescents with autism in an aquatic setting. Journal of Applied Behavior Analysis, 53(1), 305–314. 10.1002/jaba.590 31215025

[bibr13-01454455261421144] *Flannery K. B. Horner R. H. (1994). The relationship between predictability and problem behavior for students with severe disabilities. Journal of Behavioral Education, 4(2), 157–176. 10.1007/BF01544110

[bibr14-01454455261421144] Flannery K. B. O’Neill R. E. Horner R. H. (1995). Including predictability in functional assessment and individual program development. Education and Treatment of Children, 18(4), 499–509.

[bibr15-01454455261421144] Garg A. X. Hackam D. Tonelli M. (2008). Systematic review and meta-analysis: When one study is just not enough. Clinical Journal of the American Society of Nephrology, 3(1), 253–260. 10.2215/CJN.01430307 18178786

[bibr16-01454455261421144] Greer B. D. Fisher W. W. Saini V. Owen T. M. Jones J. K . (2016). Functional communication training during reinforcement schedule thinning: An analysis of 25 applications. Journal of Applied Behavior Analysis, 49(1), 105–121. 10.1002/jaba.265 26482103 PMC4860882

[bibr17-01454455261421144] Hanley G. P. Iwata B. A. McCord B. E. (2003). Functional analysis of problem behavior: A review. Journal of Applied Behavior Analysis, 36(2), 147–185. 10.1901/jaba.2003.36-147 12858983 PMC1284431

[bibr18-01454455261421144] Hanley G. P. Jin C. S. Vanselow N. R. Hanratty L. A. (2014). Producing meaningful improvements in problem behavior of children with autism via synthesized analyses and treatments. Journal of Applied Behavior Analysis, 47(1), 16–36. 10.1002/jaba.106 24615474

[bibr19-01454455261421144] Horner R. H. Carr E. G. Strain P. S. Todd A. W. Reed H. K. (2002). Problem behavior interventions for young children with autism: A research synthesis. Journal of Autism and Developmental Disorders, 32(5), 423–446. 10.1023/a:1020593922901 12463518

[bibr20-01454455261421144] *Huffman R. W. Sainato D. M. Curiel E. S. (2016). Correspondence training using special interests to increase compliance during transitions: An emerging technology. Behavior Analysis in Practice, 9(1), 25–33. 10.1007/s40617-015-0100-1 27606230 PMC4788650

[bibr21-01454455261421144] *Iadarola S. Shih W. Dean M. Blanch E. Harwood R. Hetherington S. Mandell D. Kasari C. Smith T. (2018). Implementing a manualized, classroom transition intervention for students with ASD in underresourced schools. Behavior Modification, 42(1), 126–147. 10.1177/0145445517711437 28675941

[bibr22-01454455261421144] Iwata B. A. Dorsey M. F. Slifer K. J. Bauman K. E. Richman G. S. (1994). Toward a functional analysis of self-injury. Journal of Applied Behavior Analysis, 27(2), 197–209. 10.1901/jaba.1994.27-197 8063622 PMC1297798

[bibr23-01454455261421144] *Jessel J. Hanley G. P. Ghaemmaghami M. (2016). A translational evaluation of transitions. Journal of Applied Behavior Analysis, 49(2), 359–376. 10.1002/jaba.283 26916573

[bibr24-01454455261421144] Kamlowsky M. E. Wilder D. A. Ertel H. Hodges A. C. Colon N. Domino L. (2021). Latency-based functional analysis and treatment of elopement. Behavioral Interventions, 36(2), 329–341. 10.1002/bin.1781

[bibr25-01454455261421144] Koyama T. Wang H. T. (2011). Use of activity schedule to promote independent performance of individuals with autism and other intellectual disabilities: A review. Research in developmental disabilities, 32(6), 2235–2242. 10.1016/j.ridd.2011.05.003 21645988

[bibr26-01454455261421144] Lequia J. Machalicek W. Rispoli M. J. (2012). Effects of activity schedules on challenging behavior exhibited in children with autism spectrum disorders: A systematic review. Research in Autism Spectrum Disorders, 6(1), 480–492. 10.1016/j.rasd.2011.07.008

[bibr27-01454455261421144] Luczynski K. C. Rodriguez N. M. (2015). Assessment and treatment of problem behavior associated with transitions. In DiGennaro Reed F. D. Reed D. D. (Eds.), Autism service delivery: Bridging the gap between science and practice (pp. 151–173). Springer. 10.1007/978-1-4939-2656-5_5

[bibr28-01454455261421144] *Mace A. B. Shapiro E. S. Mace F. C. (1998). Effects of warning stimuli for reinforcer withdrawal and task onset on self-injury. Journal of Applied Behavior Analysis, 31(4), 679–682. 10.1901/jaba.1998.31-679 9891405 PMC1284159

[bibr29-01454455261421144] Matson J. L. Mahan S. Hess J. A. Fodstad J. C. Neal D. (2010). Progression of challenging behaviors in children and adolescents with autism spectrum disorders as measured by the Autism Spectrum Disorders-Problem Behaviors for Children (ASD-PBC). Research in Autism Spectrum Disorders, 4(3), 400–404. 10.1016/j.rasd.2009.10.010

[bibr30-01454455261421144] *McCord B. E. Thomson R. J. Iwata B. A. (2001). Functional analysis and treatment of self-injury associated with transitions. Journal of Applied Behavior Analysis, 34(2), 195–210. 10.1901/jaba.2001.34-195 11421312 PMC1284312

[bibr31-01454455261421144] Melanson I. J. Fahmie T. A. (2023). Functional analysis of problem behavior: A 40-year review. Journal of Applied Behavior Analysis, 56(2), 262–281. 10.1002/jaba.983 36892835

[bibr32-01454455261421144] Mitteer D. M. Peterson K. M. Crowley-Zalaket J. G. Greer B. D. (2023). Transition-related problem behavior. In Matson J. L. (Ed.), Handbook of applied behavior analysis for children with autism (pp. 255–275). Springer Nature.

[bibr33-01454455261421144] Moeyaert M. Dehghan-Chaleshtori M. Xu X. Yang P (2023) Single-case design meta-analyses in education and psychology: A systematic review of methodology. Frontiers in Research Metrics and Analytics, 8, 1190362. 10.3389/frma.2023.1190362 38025959 PMC10679716

[bibr34-01454455261421144] Moeyaert M. Ferron J. Beretvas S. Van den Noortgate W. (2014). From a single-level analysis to a multilevel analysis of single-subject experimental data. Journal of School Psychology, 52(2), 191–211. 10.1016/j.jsp.2013.11.003 24606975

[bibr35-01454455261421144] Moeyaert M. Ugille M. Ferron J. Beretvas S. Van den Noortgate W. (2013). The three-level synthesis of standardized single-subject experimental data: A Monte Carlo simulation study. Multivariate Behavioral Research, 48(5), 719–748. 10.1080/00273171.2013.816621 26741060

[bibr36-01454455261421144] Moeyaert M. Xue Y. Yang P. (2023). Individual participant data meta-analysis including moderators: Empirical validation. Journal of Experimental Education, 92(4), 723–740. 10.1080/00220973.2023.2208062

[bibr37-01454455261421144] National Institute of Health. (2024). Intellectual and developmental disabilities (IDDs). Retrieved December 28, 2024, from https://www.nichd.nih.gov/health/topics/factsheets/idds

[bibr38-01454455261421144] Perone M. Courtney K. (1992). Fixed-ratio pausing: Joint effects of past reinforcer magnitude and stimuli correlated with upcoming magnitude. Journal of the Experimental Analysis of Behavior, 57(1), 33–46. 10.1901/jeab.1992.57-33 16812647 PMC1323067

[bibr39-01454455261421144] Piazza C. C. Hanley G. P. Bowman L. G. Ruyter J. M. Lindauer S. E. Saiontz D. M. (1997). Functional analysis and treatment of elopement. Journal of Applied Behavior Analysis, 30(4), 653–672. 10.1002/jaba.376 9433790 PMC1284082

[bibr40-01454455261421144] Piazza C. C. Moes D. R. Fisher W. W. (1996). Differential reinforcement of alternative behavior and demand fading in the treatment of escape-maintained destructive behavior. Journal of Applied Behavior Analysis, 29(4), 569–572. 10.1901/jaba.1996.29-569 8995837 PMC1284011

[bibr41-01454455261421144] Pitts R. C. Hughes C. E. Williams D. C. (2019). Transitions from rich-to-lean schedules increase attack in a laboratory model of social aggression in pigeons: II. Fixed-interval schedules. Mexican Journal of Behavior Analysis, 45(2), 519–546. 10.5514/rmac.v45.i2.75582

[bibr42-01454455261421144] *Repp A. C. Karsh K. G. (1994). Hypothesis-based interventions for tantrum behaviors of persons with developmental disabilities in school settings. Journal of Applied Behavior Analysis, 27(1), 21–31. 10.1901/jaba.1994.27-21 8188561 PMC1297774

[bibr43-01454455261421144] Ryan È. Bailey A. L. Grace Y. H . (2019). Rethinking the role of transitions between activities in early childhood settings: An examination of their linguistic characteristics in two preschool classrooms. Journal of Early Childhood Literacy, 21(4), 1468798419870596. 10.1177/1468798419870596

[bibr44-01454455261421144] *Sainato D. M. Strain P. S. Lefebvre D. Rapp N. (1987). Facilitating transition times with handicapped preschool children: A comparison between peer-mediated and antecedent prompt procedures. Journal of Applied Behavior Analysis, 20(3), 285–291. 10.1901/jaba.1987.20-285 3667478 PMC1286020

[bibr45-01454455261421144] Schreibman L. Whalen C. Stahmer A. C. (2000). The use of video priming to reduce disruptive transition behavior in children with autism. Journal of Positive Behavior Interventions, 2(1), 3–11. 10.1177/109830070000200102

[bibr46-01454455261421144] *Smith L. K. C. Fowler S. A. (1984). Positive peer pressure: The effects of peer monitoring on children’s disruptive behavior. Journal of Applied Behavior Analysis, 17(2), 213–227. 10.1901/jaba.1984.17-213 6735953 PMC1307935

[bibr47-01454455261421144] Sterling-Turner H. E. Jordan S. S. (2007). Interventions addressing transition difficulties for individuals with autism. Psychology in the Schools, 44(7), 681–690. 10.1002/pits.20257

[bibr48-01454455261421144] Stokes T. F. Baer D. M. (1977). An implicit technology of generalization. Journal of Applied Behavior Analysis, 10(2), 349–367. 10.1901/jaba.1977.10-349 16795561 PMC1311194

[bibr49-01454455261421144] Strand R. C. W. Vister O. M. Eldevik S. Eikeseth S. (2021). Nature, prevalence, and characteristics of challenging behaviors in functional assessment. In Matson J. L. (Ed.), Functional assessment for challenging behaviors and mental health disorders (pp. 87–109). Springer. 10.1007/978-3-030-66270-7_5

[bibr50-01454455261421144] Tevis C. Matson J. L. (2022). Challenging behaviour in children with developmental disabilities: An overview of behavioural assessment and treatment methods. BJPsych Advances, 28(6), 401–409. 10.1192/bja.2022.59

[bibr51-01454455261421144] Tiger J. H. Hanley G. P. Bruzek J. (2008). Functional communication training: A review and practical guide. Behavior Analysis in Practice, 1(1), 16–23. 10.1007/BF03391716 22477675 PMC2846575

[bibr52-01454455261421144] Toegel F. Perone M. (2022). Effects of advance notice on transition-related pausing in pigeons. Journal of the Experimental Analysis of Behavior, 117(1), 3–19. 10.1002/jeab.730 34859444

[bibr53-01454455261421144] *Tustin R. D. (1995). The effects of advance notice of activity transitions on stereotypic behavior. Journal of Applied Behavior Analysis, 28(1), 91–92. 10.1901/jaba.1995.28-91 16795856 PMC1279793

[bibr54-01454455261421144] Van den Noortgate W. Onghena P . (2007). The aggregation of single-case results using hierarchical linear models. The Behavior Analyst Today, 8(2), 196–209. 10.1037/h0100613

[bibr55-01454455261421144] *Vasquez S. Brewer A. Leon Y. Vasquez J. (2017). The effects of advance notice on problem behavior occasioned by interruptions of an ongoing activity in a young girl with autism. Behavior Analysis in Practice, 10(4), 417–421. 10.1007/s40617-017-0187-7 29214139 PMC5711738

[bibr56-01454455261421144] Vollmer T. R. Bacotti J. K. Lloveras L. A . (2022). Extinction and differential reinforcement. In Leaf J. B. Cihon J. H. Ferguson J. L. Weiss M. J. (Eds.), Handbook of applied behavior analysis interventions for autism: Integrating research into practice (pp. 539–554). Springer. 10.1007/978-3-030-96478-8_28

[bibr57-01454455261421144] Vollmer T. R. Iwata B. A . (1992). Differential reinforcement as treatment for behavior disorders: Procedural and functional variations. Research in Developmental Disabilities, 13(4), 393–417. 10.1016/0891-4222(92)90013-v 1509180

[bibr58-01454455261421144] Wade-Galuska T. Perone M. Wirth O. (2005). Effects of past and upcoming response-force requirements on fixed-ratio pausing. Behavioural Processes, 68(1), 91–95. 10.1016/j.beproc.2004.10.001 15639389

[bibr59-01454455261421144] *Waters M. B. Lerman D. C. Hovanetz A. N. (2009). Separate and combined effects of visual schedules and extinction plus differential reinforcement on problem behavior occasioned by transitions. Journal of Applied Behavior Analysis, 42(2), 309–313. 10.1901/jaba.2009.42-309 19949517 PMC2695333

[bibr60-01454455261421144] What Works Clearinghouse. (2020). What Works Clearinghouse standards handbook (Version 4.1). National Center for Education Evaluation and Regional Assistance, Institute of Education Sciences, U.S. Department of Education. https://ies.ed.gov/ncee/wwc/handbooks

[bibr61-01454455261421144] Wilder D. A. Chen L. Atwell J. Pritchard J. Weinstein P. (2006). Brief functional analysis and treatment of tantrums associated with transitions in preschool children. Journal of Applied Behavior Analysis, 39(1), 103–107. 10.1901/jaba/2006.66-04 16602389 PMC1389600

[bibr62-01454455261421144] Wilder D. A. Zonneveld K. Harris C. Marcus A. Reagan R. (2007). Further analysis of antecedent interventions on preschoolers’ compliance. Journal of Applied Behavior Analysis, 40(3), 535–539. 10.1901/jaba.2007.40-535 17970266 PMC1986698

[bibr63-01454455261421144] Williams D. C. Hayashi Y. Brewer A. Saunders K. J. Fowler S. Pitts R. C. (2019). Transitions from rich-to-lean schedules increase attack in a laboratory model of social aggression in pigeons: I. Fixed-ratio schedules. Mexican Journal of Behavior Analysis, 45(2), 500–518. 10.5514/rmac.v45.i2.75580

[bibr64-01454455261421144] Williams D. C. Saunders K. J. Perone M. (2011). Extended pausing by humans on multiple fixed-ratio schedules with varied reinforcer magnitude and response requirements. Journal of the Experimental Analysis of Behavior, 95(2), 203–220. 10.1901/jeab.2011.95-203 21541121 PMC3047253

